# Aβ1-42-containing platelet-derived extracellular vesicle is associated with cognitive decline in Parkinson’s disease

**DOI:** 10.3389/fnagi.2023.1170663

**Published:** 2023-04-14

**Authors:** Ziyu Wang, Yuanchu Zheng, Huihui Cai, Chen Yang, Siming Li, Hong Lv, Tao Feng, Zhenwei Yu

**Affiliations:** ^1^Center for Movement Disorders, Department of Neurology, Beijing Tiantan Hospital, Capital Medical University, Beijing, China; ^2^Clinical Diagnosis Department of Beijing Tiantan Hospital, Capital Medical University, Beijing, China; ^3^China National Clinical Research Center for Neurological Diseases, Beijing, China; ^4^Beijing Neurosurgical Institute, Capital Medical University, Beijing, China

**Keywords:** Parkinson’s disease, cognitive decline, Aβ1-42, activated platelet, extracellular vesicles

## Abstract

**Background:**

Cortical amyloid deposition is a common observation in Parkinson’s disease dementia (PDD) patients. Aβ1-42 is linked to a more rapid progression of dementia. Platelets, which degranulate upon activation, are a primary source of Aβ. It has been repeatedly reported that peripheral extracellular vesicles (EVs) can partially reach the central nervous system. Thus, we speculate that activated platelet-derived Aβ1-42-containing EVs (PEV-Aβ1-42) play a crucial role in the cognitive decline of PD patients.

**Methods:**

The study included 189 participants: 66 with non-dementia PD, 73 with PDD, and 50 healthy controls. All participants underwent blood collection and clinical assessments. Twenty PD patients underwent re-examination and repeated blood collection 14 months later. A nano-scale flow cytometry assay was used to detect PEVs and PEV-Aβ1-42 using fluorescence-labeled CD62P and Aβ1-42 antibodies.

**Results:**

Parkinson’s disease dementia patients had higher PEV-Aβ1-42 concentrations than healthy controls (*p* = 0.028). The ratio of PEV-Aβ1-42 to PEV was significantly higher in PDD patients compared to those in non-dementia PD and healthy controls (*p*_PD-ND_ < 0.001, *p*_HC_ = 0.041). The PEV-Aβ1-42/PEV ratio appears to influence the odds of developing dementia (OR = 1.76, *p* < 0.001). The change in the PEV-Aβ1-42/PEV ratio was also correlated with cognitive decline over 14 months (*r* = −0.447, *p* < 0.05).

**Conclusion:**

The plasma PEV-Aβ1-42/PEV ratio may serve as a diagnostic and prognostic biomarker for PDD patients.

## Introduction

Parkinson’s disease (PD) is the most common movement disorder and the second-most common neurodegenerative disease after Alzheimer’s disease (AD) (Aarsland et al., 2021), with resting tremor, rigidity, bradykinesia, and postural instability as primary symptoms. In addition to movement disorders, PD patients suffer from a series of non-motor symptoms, with cognitive decline being one of the most notable (Yang et al., 2022). Cognitive impairment significantly affects hospitalization and mortality in PD patients (Vasconcellos and Pereira, 2015). The biomarkers for cognitive impairment in PD are crucial, for individuals who are experiencing cognitive decline are prone to be diagnosed with dementia in the coming years (Hoogland et al., 2017). α-Synuclein aggregates in limbic and neocortical regions of the brain (Lewy bodies), along with Alzheimer-type pathologies, are neuropathological hallmarks of Parkinson’s disease dementia (PDD) (Halliday et al., 2008; Irwin et al., 2013). Since molecular imaging approaches permit the detection of pathological accumulations of amyloid-β plaques (Klunk et al., 2004), substantial amyloid-β (Aβ) deposition was repeatedly reported in the brain of PDD patients (Kalaitzakis et al., 2008; Jellinger, 2009; Compta et al., 2011; Halliday et al., 2011; Palermo et al., 2019). In addition, higher Aβ scores are associated with a faster cognitive decline in PD (Compta et al., 2011). The main ingredient of senile plaques, Aβ1-42, is crucial for Aβ pathology (O'Brien and Wong, 2011; Sancesario et al., 2018). Anti-Aβ1-42 therapy has also been demonstrated to relieve the Aβ pathology (Serrano-Pozo et al., 2010).

Amyloid precursor protein (APP), α-secretase, and β-secretase, the key molecules engaged in the amyloid processing approach (Colciaghi et al., 2004), can be found in platelets. Notably, platelets are the primary peripheral source of Aβ, which is processed in a mechanism similar to neurons (Li et al., 1995; Evin et al., 2003). Research has confirmed that blood-derived Aβ can enter the brain, form Aβ-related pathologies, and induce functional deficits in neurons (Stellos et al., 2010; Bu et al., 2018; Wu et al., 2021). APP and Aβ are found in platelet α-granules which are degranulated during platelet activation (Van Nostrand et al., 1990; Shen et al., 2008). These degranulated extracellular vesicles (EV) carry specific proteins, including p-selectin (CD62P), which is considered a reliable biomarker of platelet activation (Carbone et al., 2021). Casoli et al. demonstrated that platelets release more Aβ1-42 under stimulation (Casoli et al., 2007), and PMS777, a platelet activation inhibitor, can reduce Aβ1-42 release (Yang et al., 2009). It has been reported that EV in peripheral circulation can partially cross the blood–brain barrier and enter the central nervous system (Qu et al., 2018). It is reasonable to speculate that Aβ bearing EV released by activated platelets plays a vital role in the Aβ related brain pathology.

Cerebrospinal fluid (CSF) Aβ1-42 can reliably predict cognitive deterioration of AD patients, offering a promising precedent for similar investigations in Parkinson’s disease (Johar et al., 2017). However, the invasiveness of lumbar puncture limits the accessibility of CSF use. Biomarkers derived from blood samples are obviously more accessible, yet the results are inconsistent. In 2018, a population-based longitudinal study with a mean follow-up of 14.8 years found that plasma Aβ1-42 may be a helpful biomarker for identifying patients at risk of dementia (Hilal et al., 2018). In contrast, a more recent study found that plasma Aβ1-42 expression levels in Parkinson’s disease patients were not associated with cognitive deterioration (Lin et al., 2018). There are diverse sources of plasma Aβ1-42, and it is important to evaluate the level of Aβ1-42 derived from specific sources, especially from activated platelet. Until now, little is known about the relationship between the Aβ1-42-containing activated platelet-derived EV (PEV-Aβ1-42) concentration and cognition decline in PD.

Here, we developed a nano-scale flow cytometry assay for measuring PEV-Aβ1-42 in peripheral blood. In this study, we intend to investigate the ratio of PEV-Aβ1-42 to activated platelet-derived EV (PEV) in PDD patients, PD patients without dementia (PD-ND) and healthy controls, and identify the correlations between the ratio of PEV-Aβ1-42 to PEV and cognitive status in PD patients.

## Method and materials

### Study design and subjects

From May 2021 to August 2022, 139 patients with PD were recruited from Beijing Tiantan Hospital, Capital Medical University. All PD patients met the International Parkinson and Movement Disorder Society Clinical Diagnostic Criteria (Postuma et al., 2015). The severity of cognitive impairment was assessed by the Mini-Mental State Examination (MMSE) and Montreal Cognitive Assessment (MoCA). The motor function of participants was assessed by using the Movement Disorder Society Sponsored-Unified Parkinson’s Disease Rating Scale and Hoehn and Yahr stage. Among the 139 PD patients, 73 cases meet the PDD criteria according to the 5th edition of diagnostic and statistical manual of mental disorders (DSM-5) (Carter, 2014), which is mainly used as the dementia criteria at the time of cohort initiation. 50 healthy controls were also included. 20 PD returned for clinical and cognitive examinations after a median interval of 14 (Q1–Q3 8.75–16) months since the first visit. The plasma was collected at both baseline and follow-up visits.

Exclusion criteria includes: (1) Cognitive dysfunction caused by Alzheimer’s disease, frontotemporal dementia, and other causes; (2) Secondary Parkinson’s syndrome caused by trauma, tumor, cerebral apoplexy, etc. (3) PDD generated by taking anticholinergic drugs. (4) Combining with mental illness and unable to complete the cognitive scale. The study was approved by the Ethics Committee of Beijing Tiantan Hospital, Capital Medical University, and was conducted according to the Declaration of Helsinki. Informed consent were obtained from all participants.

### Plasma sampling and processing

K2-EDTA Vacutainers (367,863, BD, Franklin Lakes, NJ, United States) were used to collect fresh blood. All samples were collected from PD patients or healthy individuals in the morning. The blood samples were thoroughly mixed by being turned upside down three to four times before centrifugation. Plasma was separated from whole blood by centrifugation at 1500 × *g* and 4°C for 10 min, and was transferred to a new tube for a second centrifugation at 12000 × *g* for 10 min at 4°C in order to eliminate potential cell debris. Then, the supernatant was transferred to another polypropylene tube and preserved at −80°C before analysis. The reference plasma was equally pooled with plasma samples from 34 PD subjects.

Extracellular vesicles-depleted plasma was generated with an extra ultracentrifugation at 150,000 × *g* and 4°C for 1.5 h. Only the top layer supernatant was collected.

### Western blotting

Five μl plasma was mixed with 15 μl RIPA (C1053+, APPLYGEN, Beijing, China) lysis buffer on ice for 10 min. Lysates were spun down (12,000 × *g*, 10 min, 4°C). The total protein concentration was determined using Bicinchoninic Acid Assay (23,225, Life Technologies, Eugene, United States). Plasma-extracted proteins (30 μg) were separated by using 10% ExpressPlus™ PAGE Gel (M01010C, Genscript, Nanjing, China) and transferred to polyvinylidene fluoride (PVDF) blotting membranes (IPVH00010, EMD Millipore Corporation, Billerica, United States). The PVDF membranes were blocked with 5% non-fat milk and incubated with the following antibodies: polyclonal anti-Alix (SAB5700777, EMD Millipore Corporation, Billerica, United States, 1:1000) and monoclonal anti-*β*-actin (ab8226, Abcam, Cambridge, United Kingdom, 1:1000) overnight at 4°C. The immunoreactive bands were visualized by a chemiluminescence kit (WBKLS0500, EMD Millipore Corporation, Billerica, United States) and Bio-Rad Chemidoc XRS^+^ Imager.

### Purification of CD62P-positive EVs in plasma

CD62P-positive EVs were immunoprecipitated by anti-CD62P (sc-19,672, Santa Cruz Biotechnology, Dallas, United States) and protein A/G agarose beads (sc-2003, Santa Cruz Biotechnology, Dallas, United States). Briefly, total plasma EVs were isolated using an ExoQuick PLUS Exosome Purification Kit (EQPL10A-1, SBI System Biosciences, Palo Alto, United States) according to the manufacturer’s instructions. Next, 2 μg anti-CD62P antibody was mixed with the resuspended EV pellet for overnight at 4°C with continuous rotation. Then, the protein A/G agarose beads were added to the antibody-protein complex and incubated at 4°C for 3 h. Finally, the EVs were eluted from the beads by using 70 μl 0.1 M glycine (pH = 3) buffer, which was balanced with 5 μl 1 M Tris buffer (pH = 7). The samples were preserved at −80°C before loading to copper grids for transmission electron microscopy imaging.

### Transmission electron microscopy

Five μl CD62P-positive EVs were loaded on the 200-mesh copper grids and stained with filtered 1% uranium acetate for 2 min. Contact the grid edge with absorbent paper to remove any excess uranium acetate solution. Rinse the grid quickly with a drop of water for another 2 min. Let the grid dry for 5 min at room temperature. Then, place the grid in the grid box for transmission electron microscopy inspection at 80 kv using a Hitachi H-7650 platform.

### Extracellular vesicles analysis with CytoFLEX flow cytometry

Fluorophore-conjugated antibodies were prepared using Zenon IgG labeling kits from Invitrogen/Life Technologies. In particular, the Zenon™ Alexa Fluor 488 mouse IgG_1_ labeling kit (Z25002, Life Technologies, Eugene, United States) was used to label the monoclonal antibody (sc-19,672, Santa Cruz Biotechnology, Dallas, United States) against CD62P. Rabbit anti-*β*-amyloid 1–42 polyclonal antibody (AB5078P, EMD Millipore Corporation, Billerica, United States) was labeled using the Zenon™ Alexa Fluor 647 rabbit IgG labeling kit (Z25308, Life Technologies, Eugene, United States). Briefly, 5 μl component A from the labeling kit was mixed with 1 μg antibody for 30 min at room temperature. Afterward, 5 μl Component B was added to the mixture for another 15 min incubation in a light-protected environment. Five μl of plasma from each subject was incubated with 0.1 μg of fluorophore-conjugated antibody for each target for 30 min at room temperature. After the reaction, the mixtures were 1:60 diluted with 0.22 μm filtered PBS before loading to the high-sensitive flow cytometer, CytoFLEX S (Beckman Coulter, Milano, Italy).

A Violet Side Scatter Hight (VSSC-H) detection mode was selected for the detection of small vesicles ranging under 500 nm. A low flow rate at 10 μl/min was used to acquire the fluorescence-labeled EVs from each sample. The gates were set up according to the IgG isotype control and the blank control. An ApogeeMix beads kit (cat # 1493, Apogee, Parkville, Australia) containing fluorescent polystyrene (PS) beads with wavelengths of 110 nm and 500 nm was used as a standard marker.

CD62P single-positive events and CD62P/Aβ1-42 dual-positive events were counted for further analysis. The ratio of ADPEV-Aβ1-42 to ADPEV is calculated using the concentration of CD62P and Aβ1-42 dual positive EV to CD62P positive EV.

### Statistical analysis

All statistical analyses were performed using R software (version 4.0.4) and Prism (version 8.0). The normality was determined by the Kolmogorov–Smirnov test. When the variables followed a Gaussian distribution, the two-tailed t-test was used to compare the data, and multiple comparisons were made using analysis of variance (ANOVA). The Mann–Whitney test was used to compare data for variables that did not follow a normal distribution, and the Kruskal-Wallis test was employed when there were more than two groups. To compare dichotomous variables between groups, the χ^2^ test was utilized. Multifactorial logistic regression analyses were performed to estimate demographic characteristics, clinical features and the ratio associated with the incidence of dementia in PD patients. The Spearman rank correlation test was employed to investigate the relationships between demographic characteristics, clinical features and the ratio. Significant was defined as *p* < 0.05.

## Result

### Demographic and clinical features

A total of 139 PD patients and 50 health controls (HC) were included in this study. Based on the presence or absence of dementia, PD patients were further classified as 66 PD-ND and 73 PDD. The demographic and clinical data of the subjects were listed in Table 1. There were no significant differences in the ratios of males to females among the PDD, PD-ND, and HC groups. The PDD group has elder participants, longer duration, and more severe movement disorders as expected, as it includes only cognitively severely declined participants. The *p-*value for disease duration between PD-ND and PDD is 0.002, and the performance for Hoehn and Yahr stage, Movement Disorder Society Sponsored-Unified Parkinson’s Disease Rating Scale III, Mini-Mental Status Examination and Montreal Cognitive Assessment in PDD patients are all significantly worse than those in PD-ND patients (*p* < 0.001).

**Table 1:** Basic clinical and demographic information of healthy control and Parkinson’s disease patients.

	HC	PD-ND	PDD	*p*			
	*n* = 50	*n* = 66	*n* = 73	Overall	HC vs. PD-ND	HC vs. PDD	PD-ND vs. PDD
Age^a^	62 [59, 67]	60.8 ± 9.2	67.5 ± 8.2	<0.001***	>0.999	0.002**	<0.0001***
Gender (Female: Male)^b^	22: 28	27: 39	37: 36	0.509	0.708	0.587	0.307
H-Y off^b^	—	2.5 [2.0;3.0]	3.0 [2.5;4.0]	<0.001***	—	—	<0.001***
MDS-UPDRS-III^c^	—	32.0 [25.0;42.0]	45.0 ± 16.0	<0.001***	—	—	<0.001***
Duration^d^	—	5.00 [3.00;8.00]	7.00 [5.00;10.0]	0.007**	—	—	0.002**
MMSE^d^	—	28.0 [27.0;30,0]	23.0 [18.5;26.0]	<0.001***	—	—	<0.001 ***
MoCA^d^	—	26.0 [23.0;28.0]	16.0 [12.5;19.0]	<0.001***	—	—	<0.001***
Ratio^e^	3.80 [1.98;5.10]	3.59 [2.23;4.69]	5.11 [2.94;6.20]	0.0003***	>0.726	0.041*	0.0002***

Data are presented as *n* (%) for categorical variables or mean ± S.D./median (interquartile range) values for continuous variables. HC, healthy controls; PD-ND, Parkinson’s disease individuals with non-dementia; PDD, Parkinson’s disease dementia; H-Y off, Hoehn and Yahr stage in off period; MDS-UPDRS, Movement Disorder Society Sponsored-Unified Parkinson’s Disease Rating Scale; MMSE, Mini-Mental Status Examination; MoCA, Montreal Cognitive Assessment; Ratio, ratio of activated platelet-derived EV containing Aβ1-42 to activated platelet-derived EV.^a^Age was compared using analysis of variance (ANOVA);^b^Gender (Female: Male) was compared using χ^2^ test;^c^MDS-UPDRS-III was compared using two-tailed *t*-test; ^d^H-Y off, duration, MMSE and MoCA were compared using Mann–Whitney test; ^e^Ratio was compared using Kruskal–Wallis test. Statistically significant, *p < 0.05; **p < 0.01, ***p < 0.001.

### Analysis of activated platelet-derived EVs

**Figure 1:**
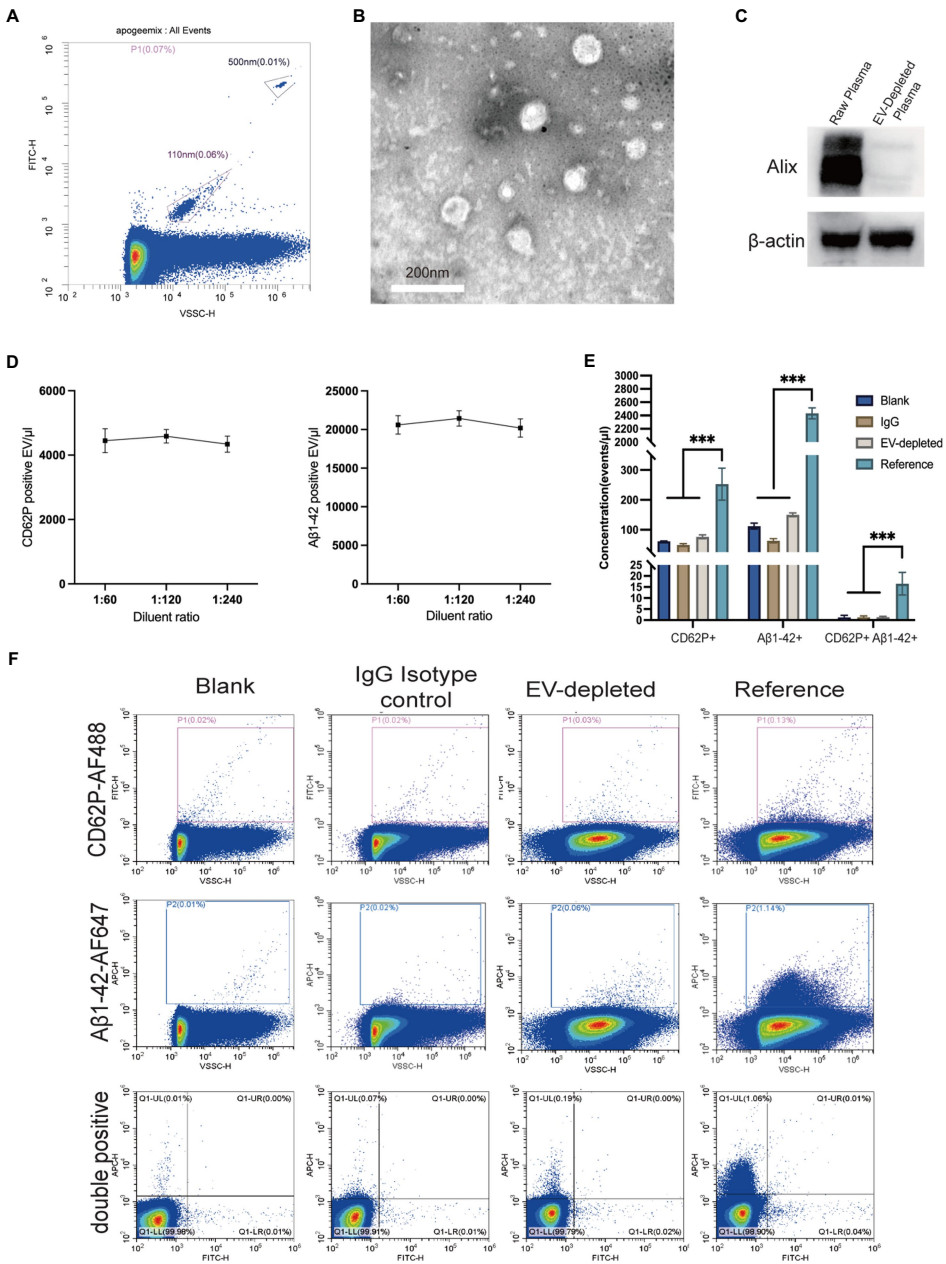
Characterization and nano-scale flow cytometry analysis of EVs in plasma. **(A)** Density plot measurement of ApoggeeMix standard beads ranging from 110 nm to 500 nm. **(B)** Transmission electron microscopy image of CD62P positive EV (scale bar = 200 nm). **(C)** The accuracy of EV detection was assessed by using a linear dilution strategy, with the dilution of 1:60, 1:120, and 1:240 for both CD62P and Aβ1-42 labeling. **(D)** Western blot verified the success of plasma EV depletion reflected by Alix levels. **(E)** Representative images of CD62P and Aβ1-42 positive EV gating in accordance with negative controls including blank control, IgG isotype control, and EV-depleted plasma control. **(F)** Signal noise ratios of CD62P and Aβ1-42 positive EV labeling assays. ***p < 0.001.

The ApogeeMix beads including FITC-labeled polystyrene beads (500 nm and 110 nm) were analyzed by using the VSSC mode of Cytoflex S platform in order to test the instrument performance. As presented in Figure 1A, the VSSC mode of Cytoflex S platform can at least detect EVs ranging from 110 to 500 nm. The CD62P-positive EVs were characterized by transmission electron microscopy (Figure 1B). Western blot analysis of Alix levels in plasma indicated the success of EV depletion by ultracentrifugation (Figure 1C). Additionally, the accuracy of EV detection was analyzed by linearity-dilution strategy. Briefly, the CD62P and Aβ1-42 antibody labeled reference plasma was diluted at the ratios of 1:60, 1:120, and 1:240 in PBS, and subjected to the Cytoflex S platform. The recovery ratios of the dilution tests were 103.1% ± 4.7% (1:120 dilution) and 97.5% ± 5.7% (1:240 dilution) for CD62P labeling, 104.1% ± 4.8% (1:120 dilution) and 98.1% ± 5.7% (1:240 dilution) for Aβ1-42 labeling (Figure 1D). The signal-noise ratio (S/N ratio) of CD62P and Aβ1-42 positive EV labeling assay was assessed by using reference plasma, no plasma control (blank), IgG isotype control and EV-depleted plasma control (Figures 1E,F). As shown in Figure 1F, the S/N ratios are 4.8, 23.9, and 10.6 for CD62P+ EV, Aβ1-42+ EV, CD62P and Aβ1-42 double positive EV assessments.

### Univariate analysis of Aβ1-42 expression in plasma activated platelet-derived EVs

**Figure 2:**
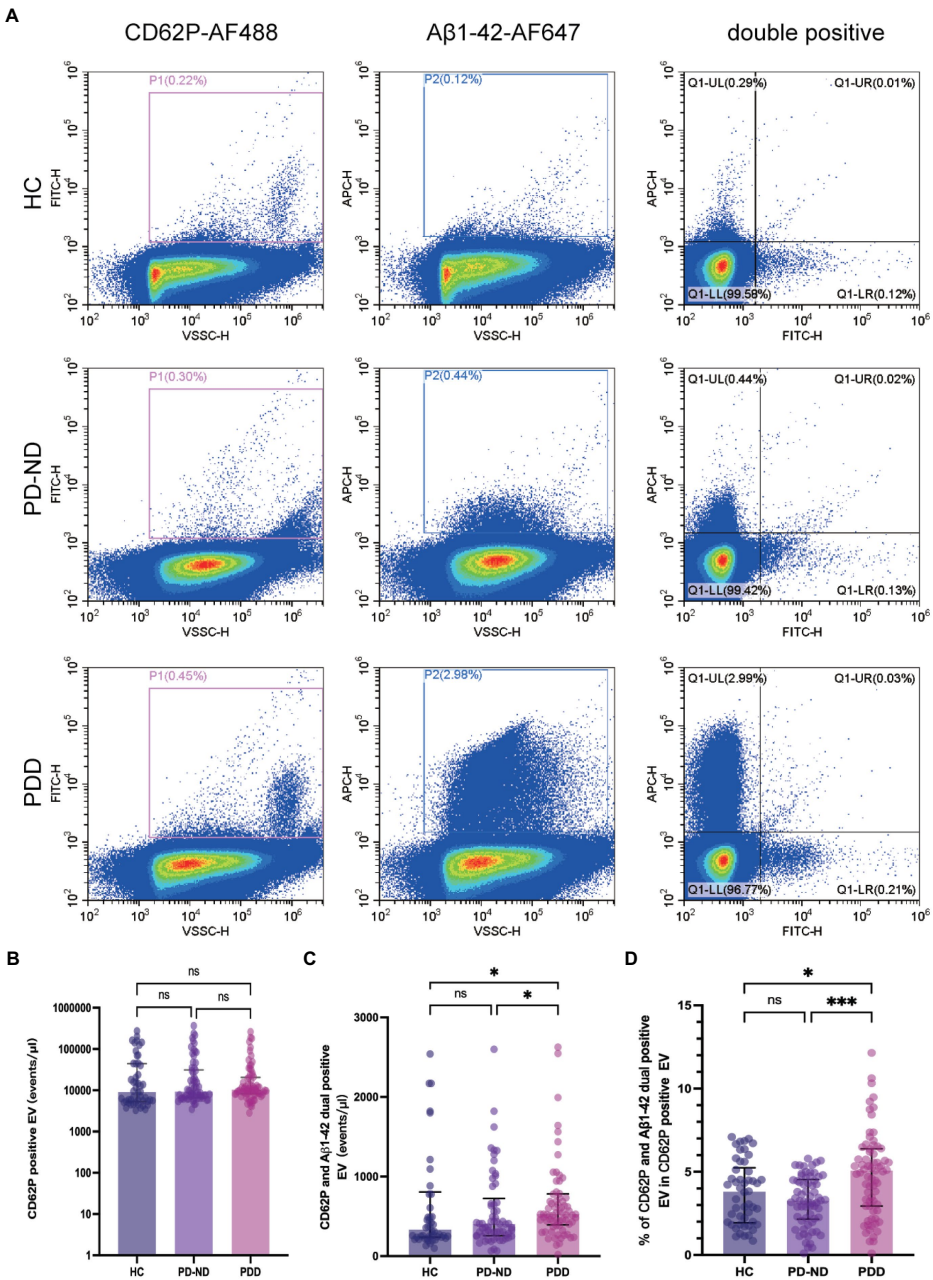
Plasma CD62P and Aβ1-42 positive EV concentrations in patients with PD and healthy controls. **(A)** Representative plots of plasma CD62P and Aβ1-42 positive EVs in HC, PD-ND and PDD patients. Comparisons of plasma **(B)** CD62P positive EV concentrations, **(C)** CD62P and Aβ1-42 double positive EV concentrations, and **(D)** the ratio of CD62P and Aβ1-42 double positive EV to CD62P positive EV concentrations in HC, PD-ND and PDD patients. *p < 0.05, ***p < 0.001.

An example of plasma EVs populations for CD62P-FITC and Aβ1-42-APC labeling are presented in Figure 2A. The concentrations of CD62P and Aβ1-42 positive EVs in the samples from 50 HC, 66 PD-ND and 73 PDD subjects are presented in Table 2 and Figures 2B–D. There was no statistical difference in the concentration of plasma CD62P positive EVs among the three groups (Table 2 and Figure 2B), but the concentration of CD62P and Aβ1-42 double positive EVs was significantly higher in PDD patients than those in HC subjects (Figure 2C, *P* = 0.028). The ratio of double positive EV to CD62P single positive EV concentrations in the PDD group was significantly higher than those in both HC and PD-ND groups (Table 2 and Figure 2D, *p*_ratio HC vs. PDD_ = 0.041 and *p*_ratio PD-ND vs. PDD_ < 0.001).

**Table 2:** Concentrations for platelet-derived EV as measured by flow cytometry.

	HC	PD-ND	PDD	*p*			
	*n* = 50	*n* = 66	*n* = 73	Over all	HC vs. PD-ND	HC vs. PDD	PD-ND vs. PDD
CD62P+ EV (events/μl)^a^	9,070 [5,333; 44,269]	9,268 [6,635;31,183]	10,403 [8,246; 20,552]	0.409	0.941	0.581	>0.999
CD62P and Aβ1-42 double positive EV (events/μl)^a^	333 [238; 805]	402 [256; 724]	526 [393; 782]	0.013*	>0.999	0.028*	0.047*
Ratio^a^	3.80 [1.98; 5.10]	3.59 [2.23; 4.69]	5.11 [2.94; 6.20]	0.0003***	>0.726	0.041*	0.0002***

Data are presented as median (interquartile range) values for continuous variables. HC, healthy controls; PD-ND, Parkinson’s disease individuals with non-dementia; PDD, Parkinson’s disease dementia; Ratio, ratio of activated platelet-derived EV containing Aβ1-42 to activated platelet-derived EV. ^a^These biomarkers were compared using Kruskal–Wallis test. Statistically significant, *p < 0.05; ***p < 0.001.

### Multifactorial logistic regression analyses for association of dementia status in PD patients

**Figure 3:**
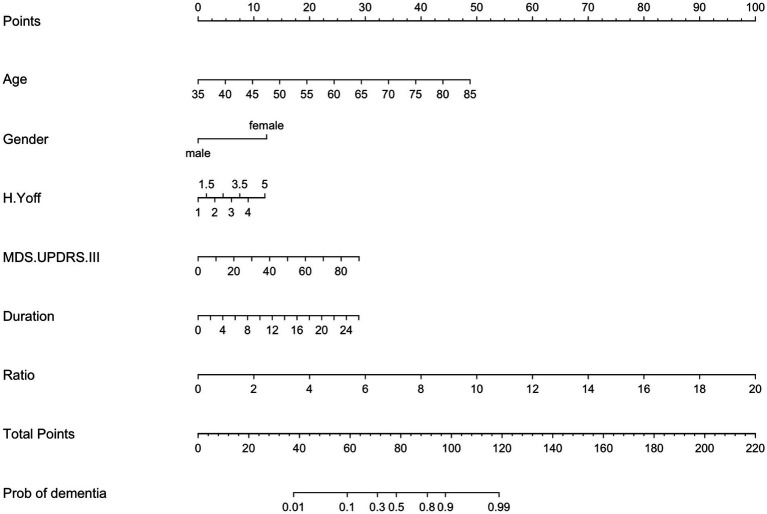
Nomogram of a multifactor logistic regression model. H.Y off, Hoehn and Yahr stage in off period; MDS.UPDRS III, Movement Disorder Society Sponsored-Unified Parkinson’s Disease Rating Scale III; MMSE, Mini-Mental Status Examination; MoCA, Montreal Cognitive Assessment; Ratio, the ratio of activated platelet-derived EV containing Aβ1-42 to total activated platelet-derived EV.

Next, we studied the association of PEV-Aβ1-42/PEV ratios and dementia status of PD patients with the control of age, gender, disease duration, H-Y stage in the off period, MDS-UPDRS-III using multifactorial logistic regression analyses (Table 3 and Figure 3). The H-Y stage in the off period does not appear to be associated with the dementia status of PD patients (OR = 1.40, 95% CI 0.63–3.15, *p* > 0.05). However, the results showed higher odds of dementia status in PD patients with advanced age (OR = 1.12, 95% CI 1.05–1.18, *p <* 0.001), longer PD duration (OR = 1.13, 95% CI 1.00–1.29, *p <* 0.05), severer movement disorders (MDS-UPDRS-III: OR = 1.04, 95% CI 1.00–1.08, *p <* 0.05), and a higher PEV-Aβ1-42/PEV ratio (OR = 1.73, 95% CI 1.34–2.32, *p <* 0.001). Among all the factors mentioned above, prior odds of developing dementia appear to be influenced by the PEV-Aβ1-42/PEV ratio. Notably, male PD patients had lower odds of developing dementia in our study (OR = 0.25, 95% CI 0.09–0.68, *p <* 0.01, Table 3 and Figure 3).

**Table 3:** Multifactorial logistic regression analyses for association of dementia status in PD patients.

	B	S.E	Wald	OR (95% CI)	*p*
Age, y	0.111	0.029	1.12 (1.05, 1.18)	<0.001***	0.111
Gender (male)	−1.393	0.515	0.25 (0.09, 0.68)	0.004**	−1.393
H-Y off	0.339	0.412	1.40 (0.63, 3.15)	0.408	0.339
MDS-UPDRS-III	0.036	0.019	1.04 (1.00, 1.08)	0.048*	0.036
Duration, y	0.126	0.065	1.13 (1.00, 1.29)	0.041*	0.126
Ratio	0.567	0.141	1.76 (1.34, 2.32)	<0.001***	0.567

H-Y off, Hoehn and Yahr stage in off period; MDS-UPDRS, Movement Disorder Society Sponsored-Unified Parkinson’s Disease Rating Scale; Ratio, ratio of activated platelet-derived EV containing Aβ1-42 to activated platelet-derived EV; B, regression coefficient; S.E, standard error; OR, odds ratio; CI, confidence interval. *Statistically significant, *p* < 0.05; **p < 0.01, ***p < 0.001.

### Correlation of the ratio with clinical features in PD patients

**Figure 4:**
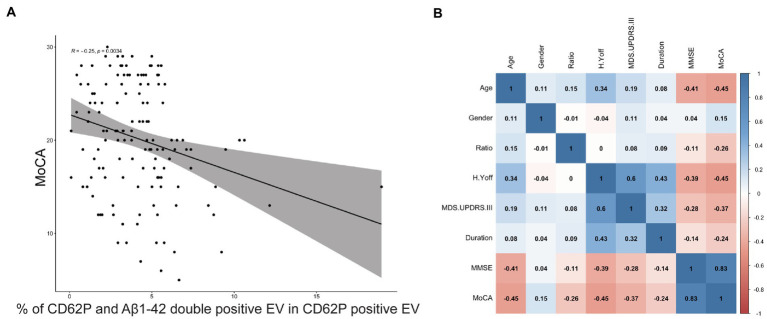
Correlation between the ratio of PEV-Aβ1-42 to PEV and the clinical characteristics of PD patients. The 95% confidence range is indicated by shaded areas. *r* = −0.25, *p* = 0.0034. MoCA, Montreal Cognitive Assessment. **(A)** Correlation between the ratio of PEV-Aβ1-42 to PEV and the MoCA scale scores of PD patients. **(B)** Correlation between demographic characteristics, clinical features and the ratio of PEV-Aβ1-42 to PEV in PD patients. The 95% confidence range is indicated by shaded areas, and correlation coefficients *r* between risk factors are shown in black font. MMSE, Mini-Mental Status Examination; MoCA, Montreal Cognitive Assessment; H.Y off, Hoehn and Yahr stage in off period; MDS.UPDRS III, Movement Disorder Society Sponsored-Unified Parkinson’s Disease Rating Scale III; Ratio, the ratio of PEV-Aβ1-42 to PEV.

To further investigate the association of PEV-Aβ1-42/PEV ratio with clinical features of PD patients including the MoCA scale, we performed Spearman linear correlation analysis. The ratio showed a significant negative association with MoCA (correlation coefficient *r* = −0.25, *p <* 0.01, Figure 4A). However, the ratio did not significantly correlate with age, gender, disease duration, H-Y stage in the off period, MMSE, or MDS-UPDRS III in the PD patients (Figure 4B).

### Correlation of the changes in the PEV-Aβ1-42/PEV ratio and MoCA scale over 14 months

**Figure 5:**
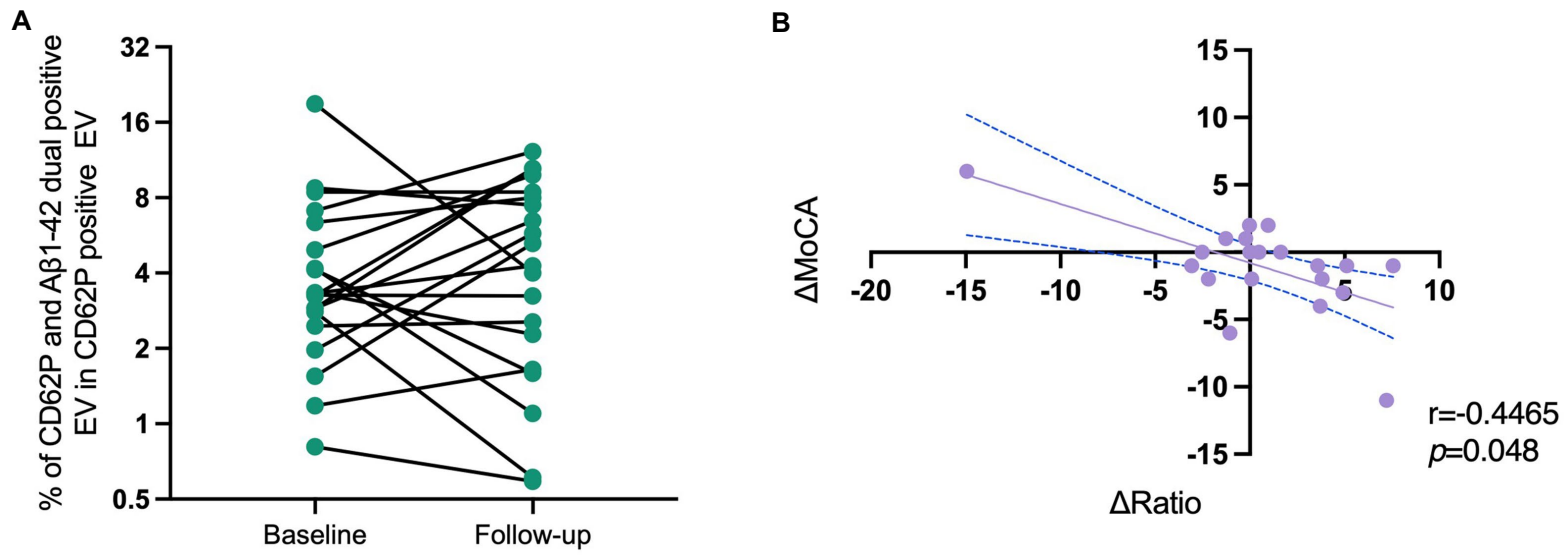
Correlation of the changes in PEV-Aβ1-42/PEV ratios and MoCA scale scores of PD patients in a longitudinal cohort. **(A)** The change of PEV-Aβ1-42/PEV ratios from baseline to follow-up. **(B)** The correlations of ΔMoCA and Δratio in PD patients. *r* = −0.447, *p* = 0.048. MoCA, Montreal Cognitive Assessment; Ratio, ratio of activated platelet-derived EV containing Aβ1-42 to activated platelet-derived EV.

There are 20 PD patients who underwent a second blood collection and clinical assessments 14 (Q1–Q3 8.75–16) months after the baseline visit. We assessed the PEV-Aβ1-42/PEV ratio again, and found that most of the PD patients (15/20) revealed a trend of increasing PEV-Aβ1-42/PEV ratios (Figure 5A). In addition, linear correlation analysis showed that the change of PEV-Aβ1-42/PEV ratios was significantly correlated with a decline in MoCA scale (Figure 5B, correlation coefficient *r* = −0.447, *p* = 0.048).

## Discussion

In this study, 189 individuals who were PDD, PD-ND patients and healthy controls were included. We examined the plasma PEV-Aβ1-42 concentrations and the PEV-Aβ1-42/PEV ratios by using a nano-scale flow cytometry platform which enables the identification of fluorescence-labeled EVs ranging from 110 nm to 500 nm. Our results indicated that the PEV-Aβ1-42/PEV ratio was significantly increased in PDD patients than in PD-ND patients. Moreover, our findings showed that prior odds of developing dementia appear to be influenced by the PEV-Aβ1-42/PEV ratio in PD patients other than age, gender, H-Y stage in the off period, MDS-UPDRS-III, and disease duration. Furthermore, we found a negative correlation between PEV-Aβ1-42/PEV ratio changes with the cognitive states of PD patients using a longitudinal cohort.

Both AD and PD patients can develop Aβ pathology. Aβ accumulated in the brain may originate from both the brain and the periphery. Sun et al. (2021) explored the role of blood cell-derived Aβ in AD pathogenesis using a bone marrow transplantation model. The human Aβ continuously expressed in the blood of wild-type (WT) mice transplanted with bone marrow cells from APPswe/PS1dE9 transgenic mice and caused AD phenotypes in the WT recipient mice. This finding indicated that peripheral Aβ plays an important role in AD pathology. This study, however, does not address whether Aβ crosses the blood–brain barrier as free molecules or within EVs. Few studies have explored Aβ1-42 in peripheral blood, especially in EVs, from PD patients. A similar study enriched peripheral blood-derived Aβ1-42-containing EV by an Immunomagnetic Reduction-based immunoassay and investigated the performance as a predictive biomarker of cognitive decline in PD patients. They reported no statistically significant difference in plasma EV Aβ1-42 levels between PD patients and controls (Chung et al., 2021). It is proven that PD patients with cognitive impairment have an increasing trend of plasma EV Aβ1-42 levels (Chung et al., 2021). However, previous research has yet to identify the source of peripheral Aβ1-42. It has been reported that platelet is a major source of Aβ in peripheral blood. The objective of this study is to learn the platelet-derived Aβ1-42, especially contained in EVs, as a biomarker for PDD patients.

Aβ1-42 has been shown to be located on EV membranes, and Aβ1-42-containing EV can be detected by fluorescence labeling using specific antibodies without disrupting EV structures (Tian et al., 2022). This approach would enable rapid detection of Aβ1-42 in PEV, without the need for EV enrichment. General risk factors of PDD include older than 75 years old, PD duration of more than 10 years, UPDRS >24, H-Y stage, impairment of semantic fluency, impairment on pentagon test, genetic factors (*GBA1, COMT, MAPT H1/H1, APOE4*), low education level, postural instability and cerebral small vascular disease (Aarsland et al., 2001; Vasconcellos and Pereira, 2015; Hou et al., 2022; Kwon et al., 2022). Compared to the healthy population, the risk of developing dementia in PD patients was reported to vary between 1.7 and 5.9 times (Marder et al., 1995; Aarsland et al., 2001; Hobson and Meara, 2004; de Lau et al., 2005; Perez et al., 2012). Our data shows that gender affects the risk of dementia in PD, with females at a higher risk than males, which is similar to the findings in Alzheimer’s disease patients (Barnes et al., 2005). This is in conflict with some research based on European, North American, Oceania, and Southeast Asian cohorts supporting that the male gender plays an important role in Parkinson’s disease and dementia (Cereda et al., 2016; Hoogland et al., 2019). This discrepancy may be due to differences in race and environment as well as sampling error. Additionally, disease duration is a key PDD risk factor (Aarsland and Kurz, 2010). The research focused on the relationship between the motor subtype of Parkinsonism and incident dementia reveals aggravation of movement disorder associated with accelerated cognitive decline (Alves et al., 2006), which is in consistent with our findings.

Univariate analysis of PEV suggested that platelet activation is not more prevalent in PDD patients compared to those in PD-ND patients, however, the PEV-Aβ1-42 levels in plasma are significantly increased in PDD patients. Related studies on platelet activation in PD and PDD patients are lacking. The mechanism of function for the increased PEV-Aβ1-42 levels in PDD has not been well understood. Different stimuli could activate platelets in different pathways, and molecules stored in granules vary according to different environmental influences and stimuli (Coppinger et al., 2007; Italiano et al., 2008; Milioli et al., 2015). Main platelet activation pathways are mediated by the binding of ADP to P2Y1 and P2Y12, the binding of TxA2 to TP and the binding of thrombin to PARS (Ferrer-Raventós and Beyer, 2021). A proteomic study based on PEV from healthy individuals reveals that APP is abundant in ADP-induced PEV and is also involved in platelet degranulation (Milioli et al., 2015). However, no study has reported the mechanism of action related to platelet activation in PD and PDD patients, which is important for further investigation.

Moreover, the results from a longitudinal sub-cohort demonstrated a negative correlation between the increase of PEV-Aβ1-42/PEV ratio and cognitive decline in PD. The ratio of PEV-Aβ1-42 to PEV may serve as a predictor for the development of dementia in PD. Notably, the MoCA scale scores elevated in two PD patients at the follow-up visit compared to the performance at the baseline visit, along with the PEV-Aβ1-42/PEV ratio increased. There is no sufficient evidence suggesting that the cognitive decline of PD patients can be reversed. One possible explanation is that there is assessment bias between different clinicians. However, assessment bias does not explain the decrease of plasma PEV-Aβ1-42/PEV ratio over time. It is possible that cognitive function can be improved in a small number of PD patients during specific disease stages, which can be reflected by the plasma PEV-Aβ1-42/PEV ratio. However, this point of view obviously needs more work and evidence to support it.

There are a series of methods including cognitive, mental, or physical training (CMPT), non-invasive brain stimulations (NIBS), drugs, or nutrients that are reported to postpone cognitive decline (Brioschi Guevara et al., 2021). Our work suggests that the suppression of PEV-Aβ1-42 secretion may be a new therapy method to improve cognitive function.

There are some limitations in this work. First, further work should be done to determine whether the biomarker we discovered is also positive for other types of dementia including Alzheimer’s disease and dementia with Lewy body. Second, the interval from baseline to the first visit was relatively short, and the sample size that was available in the follow-up cohort was limited. The current longitudinal cohort still needs further follow-up and sample size expansion.

In conclusion, the current study demonstrated that the plasma PEV-Aβ1-42 level is significantly increased in PDD patients. The plasma PEV-Aβ1-42/PEV ratio may serve as a potential biomarker for cognitive decline in PD.

## Data availability statement

The original contributions presented in the study are included in the article/supplementary material, further inquiries can be directed to the corresponding authors.

## Ethics statement

The studies involving human participants were reviewed and approved by the Ethics Committee of Beijing Tiantan Hospital, Capital Medical University. The patients/participants provided their written informed consent to participate in this study.

## Author contributions

ZW and YZ conceptualized, organized, and executed the research, designed, executed, and critically revised the statistical analysis, wrote the first draf of the manuscript, and caritically revised the manuscript. HC organized and excuted the research. CY and SL executed the research. HL, TF, and ZY: organized the research, reviewed and critically revised the statistical analysis and manuscript. All authors contributed to the article and approved the submitted version.

## Funding

This study was supported by the National Natural Science Foundation of China (Grant Numbers 82071422, 81901151, and 82020108012) and Beijing Municipal Natural Science Foundation (Grant Number 7212031 and 7232013).

## Conflict of interest

The authors declare that the research was conducted in the absence of any commercial or financial relationships that could be construed as a potential conflict of interest.

## Publisher’s note

All claims expressed in this article are solely those of the authors and do not necessarily represent those of their affiliated organizations, or those of the publisher, the editors and the reviewers. Any product that may be evaluated in this article, or claim that may be made by its manufacturer, is not guaranteed or endorsed by the publisher.

## Abbreviations

PD: Parkinson’s disease
PDD: Parkinson’s disease dementia
Aβ: amyloid-β
APP: amyloid precursor protein
EV: extracellular vesicles
PEV: activated platelet-derived extracellular vesicles
PEV-Aβ1-42: activated platelet-derived EV containing Aβ1-42
MMSE: Mini-Mental State Examination
MOCA: Montreal Cognitive Assessment
PD-ND: Parkinson’s disease individuals with non-dementia
HC: healthy controls
OR: odds ratio
H-Y: Hoehn and Yahr stage
MDS-UPDRS: Movement Disorder Society Sponsored-Unified Parkinson’s Disease Rating Scale.
